# SEM-PLS Analysis of Inhibiting Factors of Cost Performance for Large Construction Projects in Malaysia: Perspective of Clients and Consultants

**DOI:** 10.1155/2014/165158

**Published:** 2014-02-13

**Authors:** Aftab Hameed Memon, Ismail Abdul Rahman

**Affiliations:** Faculty of Civil and Environmental Engineering, Universiti Tun Hussein Onn Malaysia, Parit Raja, 86400 Batu Pahat, Johor, Malaysia

## Abstract

This study uncovered inhibiting factors to cost performance in large construction projects of Malaysia. Questionnaire survey was conducted among clients and consultants involved in large construction projects. In the questionnaire, a total of 35 inhibiting factors grouped in 7 categories were presented to the respondents for rating significant level of each factor. A total of 300 questionnaire forms were distributed. Only 144 completed sets were received and analysed using advanced multivariate statistical software of Structural Equation Modelling (SmartPLS v2). The analysis involved three iteration processes where several of the factors were deleted in order to make the model acceptable. The result of the analysis found that *R* ^2^ value of the model is 0.422 which indicates that the developed model has a substantial impact on cost performance. Based on the final form of the model, contractor's site management category is the most prominent in exhibiting effect on cost performance of large construction projects. This finding is validated using advanced techniques of power analysis. This vigorous multivariate analysis has explicitly found the significant category which consists of several causative factors to poor cost performance in large construction projects. This will benefit all parties involved in construction projects for controlling cost overrun.

## 1. Introduction

Poor cost performance in construction projects is a well-known element in resulting huge amount of cost overrun as faced by construction industry globally. The cost overrun is very dominant in both developed and developing countries [1]. It affects both physical and economic development for the country and thus, it is important to ensure construction projects are completed within the estimated cost. Numerous worldwide researches have been conducted to understand cost performance of construction projects. Meng [2] also investigated UK construction and found that 26 (25.2%) of 103 investigated projects faced overrun. Case study conducted by Chang [3] on four projects in USA found that the entire projects facing cost overrun ranged from 12.3% to 51.3% with an average of 24.8% of the contract sum. Similarly, Žujo et al. [4] studied 92 traffic structures in Slovenia and found that the construction cost exceeded 51% of the budgeted cost.

Compared to the developed countries, the cost overrun experienced in developing countries is more serious. In India, a study on 290 projects with a contract sum of 270,568 million Indian rupees faced a total of 200,024 million Indian rupees of cost overrun where an average each project faced 73% exceeding the estimated cost as cited by [5]. In Korea, Lee [6] examined 161 projects which included 138 road projects, 16 rail projects, 2 airport, and 5 port projects. His findings indicate that 95% of road projects faced 50% cost overrun; all the rail projects also faced 50% cost overrun while 2 airports projects experienced 100% cost overrun and 5 port projects experienced about 40% cost overrun. An investigation of 137 construction projects in Nigeria found that 55% of projects faced cost overrun within the range of 5% to 808% of the projects cost [7]. Northern by-pass project in Kampala, Uganda, experienced cost overrun with more than 100% while, in other study, it was found that 53% of 30 construction projects investigated faced cost overruns [8].

Likewise, Malaysian construction industry is also affected by cost overrun burdens. Khamidi et al. [9] quoted from the summary report of Malaysian Auditor General 2008 that electrification of double track rail project between Rawang and Ipoh has resulted in cost overrun of RM 1.43 billion. Endut et al. [10] in their study on 308 public and 51 private construction projects found that only 46.8% of the public projects and 37.2% of the private projects completed within the budget. Further, a survey conducted in the southern region of Peninsular Malaysia highlighted that 89% of 140 respondents mentioned that most their projects faced cost overrun [11].

Thus, cost overrun is a pertinent issue in the construction industry which needs serious attention in improving project's cost performance as the overrun is an additional burden to all parties involved in the project. It is important to identify causative factors to cost overrun in order to manage the cost performance of the projects effectively. Hence, this study focused on uncovering the inhibiting factors to cost performance of large construction projects in Malaysia. An advanced multivariate analysis method of Structural Equation Modelling (SEM) which is a graphical equivalent of a mathematical representation [12] was adopted for this analysis as it is a very effective approach in analysing cause-effect relations between factors [13].

## 2. Inhibiting Factors to Cost Performance

Occurrences of poor cost performance in construction projects are due to various factors. These inhibiting factors are referred to as cost overrun factors by many researchers. Literature reviewed on Kaming et al. [14] work indicates that major factors affecting project cost in high-rise building projects are materials cost increased by inflation, inaccurate quantity take-off, labour cost increased due to environment restriction, lack of experience on project location, lack of experience of project type, unpredictable weather conditions, and lack of experience of local regulation. In Chang [3] study, two reasons for cost increase in engineering design projects are owner request of changes in scope and additional works. While in Koushki et al. [15] study on private residential projects, the main contributors' factors are contractor-related problems, material-related problems, and owners' financial constraints, Enshassi et al. [16] studied construction projects specifying that main factors are increment of materials prices, delay in construction, supply of raw materials and equipment by contractors, fluctuations in the cost of building materials, unsettlement of local currency, project materials monopoly by some suppliers, resources constraint(funds and associated auxiliaries, not ready), lack of cost planning/monitoring during pre- and postcontract stages, improvements to standard drawings during the construction stage, design changes, and inaccurate quantity take-off.

Nawaz et al. [17] conducted a survey among constructions professionals, contractors, architects, design designers, suppliers, and subcontractors in Pakistan and identified 10 main factors which affect cost performance: corruption and bribery, political interests, poor site management, delay in site mobilization, rigid attitude by consultants, extra work without approvals, frequent changes during execution, gold plating, safety and health, and limited access to job sites. Park and Papadopoulou [18] reported that most significant causes of cost overruns in infrastructure projects experienced in Asia are contract awarded to the lowest bidder, inadequate site investigations, unforeseen site conditions, inadequate pre-construction study, and inaccurate estimates.

## 3. Conceptual Model

**Figure 1:**
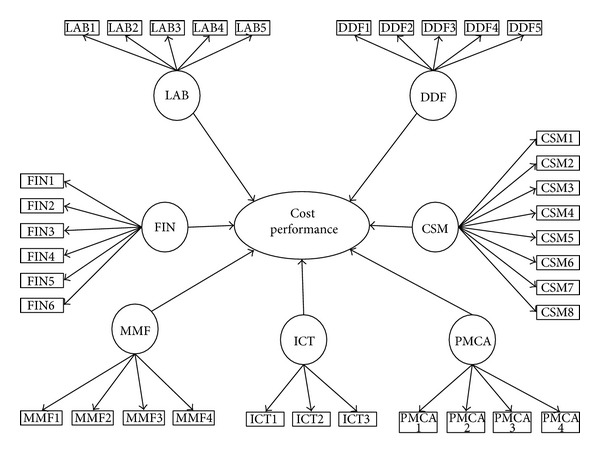
Conceptual model of cost overrun factors.

**Figure 2:**
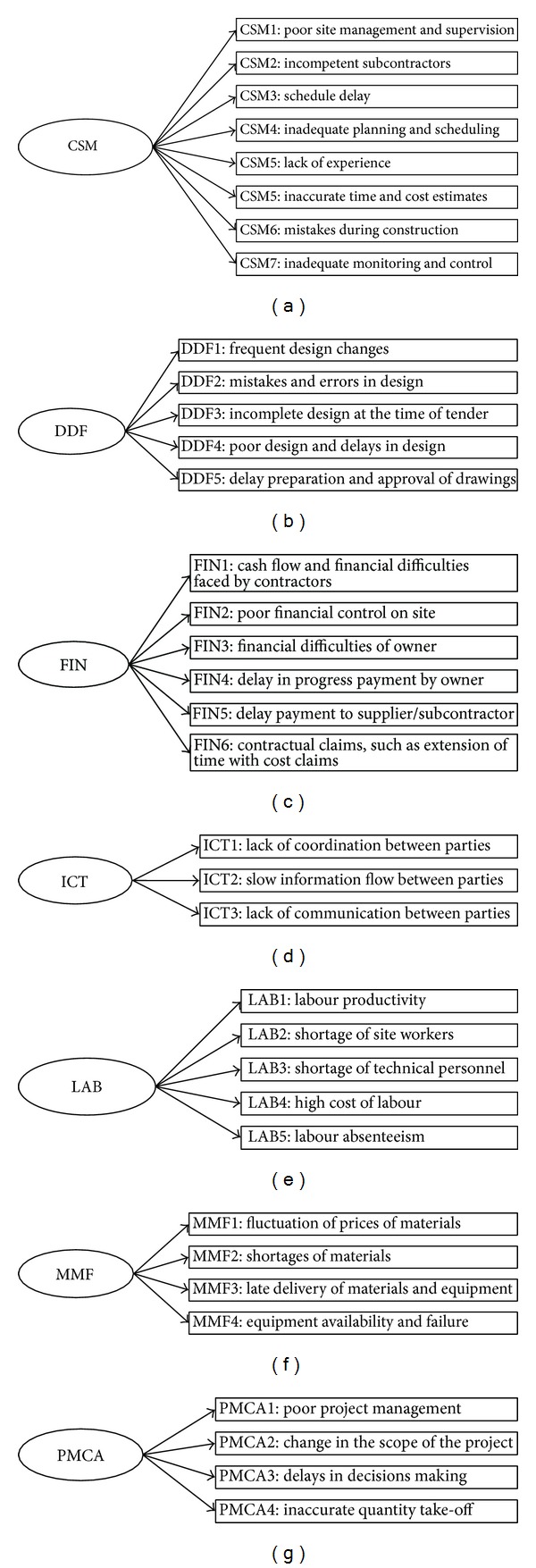
Path diagrams showing the descriptions of manifest variables.

In assessing the effect of inhibiting factors using PLS-SEM, a conceptual model is required. This model is explained in the relations between latent variables and their relative manifest variables. In this study, the conceptual model is developed based on 35 inhibiting factors (also known as manifest variables) which are grouped into 7 categories (known as exogenous latent variables) named as Contractor's Site Management Related Factors (CSM), Design and Documentation Related Factors (DDF), Financial Management Related Factors (FIN), Information and Communication Related Factors (ICT), Human Resource (Workforce) Related Factors (LAB), Nonhuman Resource Related Factors (MMF), and Project Management and Contract Administration Related Factors (PMCA). Conceptual model showing relation between LV and manifest variables is shown in Figure 1 where LVs are drawn with oval shape while rectangular shaped elements represent manifest variables. In PLS-SEM, generally the model is described by two components referred to as (1) measurement model or construct which relates manifest variables with relative LV and (2) structural model which shows the relationship between various LVs [19]. The description of each manifest variable is presented in path diagrams for each construct shown in Figures 2(a)–2(g).

## 4. Data Collection and Sampling

Method of data collection is governed by the conceptual model that was developed earlier. For this study, the data was gathered using structured questionnaire survey. The survey was conducted amongst clients and consultants involved in handling large construction projects in Malaysia. A total of 300 questionnaire forms (150 among client firms and 150 among consultant firms) were distributed in 11 states of Peninsular Malaysia. As a response, 156 completed questionnaire sets were received, of which 12 questionnaire sets were incomplete and considered inappropriate. The analysis used 144 completed questionnaire sets which are sufficient based on Hair et al. [13] rule of thumb for sample size required in PLS-SEM. Based on the completed questionnaire sets, demography of the respondents is presented in Table 1.

Table 3 shows that the participation of the consultant is very high with 92 of 100 and only 52 are clients in the survey. Majority of the respondents (68%) had working experience for more than 10 years in handling construction projects. Also, 76% of respondents have attained engineering degree. Majority of respondents are handling directorate, managerial, and engineering positions in their respective organizations. This indicates that the participants in the survey are competent and hence the collected data is considered valid.

## 5. PLS-SEM Evaluation/Analysis

The developed conceptual model was drawn in SmartPLS software [20] for simulation work in assessing the effect of manifest variables (inhibiting factors) on construction cost performance. PLS simulation of the model is carried out by calculating and assessing various parameters which include item loading, reliability, and validity tests. It involves a 2-step process as suggested by Henseler et al. [21] which involve calculating PLS model parameters separately by solving out the blocks of the measurement model and then estimating the path coefficients of a structural model [22]. Finally, overall model is validated power analysis test.

### 5.1. Measurement Model Evaluation

Measurement model evaluation is aimed to evaluate the consistency and validity of the manifest variables. Consistency evaluations are through individual manifest and construct reliability tests. While validity of the variables is tested based on convergent and discriminant validity [23], individual manifest reliability explains the variance of individual manifest relative to latent variable by calculating standardised outer loadings of the manifest variables [24]. Manifest variables with outer loading 0.7 or higher are considered highly satisfactory [21, 24]. While loading value of 0.5 is regarded as acceptable, the manifest variables with loading value of less than 0.5 should be dropped [25, 26]. Hulland [27] argued that 0.4 should be the acceptable loading value where Henseler et al. [21] suggested that manifest variable with loading values between 0.4 and 0.7 should be reviewed before elimination. If elimination of these indicators increases the composite reliability then discard or otherwise maintain the factors. Even though for this study the cut-off value taken for outer loading is 0.5, an iterative process is adopted for elimination of the manifest variables by considering Henseler et al. [21] suggestion.

Second parameter for consistency evaluations is constructed reliability where it is evaluated by two measures, that is, Cronbach's alpha and Composite Reliability (CR). Cronbach's alpha and CR indicate how well a set of manifest variables appraises a single latent construct. However, compared to Cronbach alpha, composite reliability is considered a better measure of internal consistency because it employs the standardized loadings of the manifest variables [28]. Nonetheless, the interpretation of composite reliability score and Cronbach's Alpha is the similar. Litwin [29] suggested that value of cronbach alpha should be higher than 0.7 and for composite reliability, the value of 0.7 is suggested as “modest” [13].

For the validity of the variable, the variables are tested on convergent and discriminant validities. Convergent validity is carried out by Average Variance Extracted (AVE) test on variables [28]. It determines the amount of variance captured by latent variable from its relative manifest variables due to measurement errors. Barclay et al. [30] and Hair et al. [13] argued that a minimum 50% of the variance from manifest variable should be captured by latent variables. This implies that AVE value of the construct should be greater than 0.5. Discriminant validity is carried out to confirm that the manifest variable in any construct is relevant to the designated latent variable where its cross-loading value in LV is higher than that in any other constructs [25].

Based on the above criteria, measurement model is evaluated by iterative process to discard the weak manifest variables from the developed model. Hence, a total of 3 iterations were involved in this study where each of the iterations was assessed based on the criteria and resulted in discarding 6 manifest variables.  Table 2 summarizes the first and final iterations only.

In the first iteration of Table 2, three constructs ICT, LAB, and MMF have parametric measurement above the cut-off values. While the other 4 constructs (CSM, DDF, FIN, and PMCA) have achieved satisfactory measurement values except AVE which is below 0.5. Following iterations has discarded 6 weak manifest variables in 4 of the constructs which are CSM02, CSM03, DDF04, FIN03, FIN04, and PMCA01.

Once the iteration process completed, the final model is checked for discriminant validity based on cross loading values generated from the final iteration as shown in Table 3. Cross loading of all the manifest variables has higher values on their relative latent variable as compared with other constructs as in the Table 3. This verifies that the manifest variables in each construct represent the assigned latent variable testifying the discriminant validity of the model.

### 5.2. Structural Model Assessment

Structural model assesses relationship between exogenous and endogenous latent variables through evaluating *R* ^2^ value, that is, coefficient of determination [23] and also *β* value, that is, path coefficients of the model [25]. *R* ^2^ corresponds to the degree of explained variance of endogenous latent variables [31] while *β* indicates the strength of an effect from variables to endogenous latent variables [32]. According to Cohen et al. [33, 34] for a good model, the value of *R* ^2^ of endogenous latent variable should be more than 0.26. Since *R* ^2^ value for the developed model is 0.422 which is higher than the suggested value, the model is considered to have substantial degree of explained variance of cost performance by inhibiting factors. Next step is assessing the path coefficient of all latent variables (paths) by comparing *β* values among all the paths. The highest *β* value symbolizes the strongest effect of predictor (exogenous) latent variable towards the dependent (endogenous) latent variable [35]. However, *β* value has to be tested for its significance level through *t*-value test. The test is achieved by performing nonparametric bootstrapping technique [25, 36, 37]. Bootstrapping technique computes *t*-value by creating prespecified number of samples. Hair et al. [13] suggested that acceptable *t*-values for a two-tailed test are 1.65 (significance level = 10 percent), 1.96 (significance level = 5 percent), and 2.58 (significance level = 1 percent). In this study, bootstrapping generated 5000 samples and these samples are used to compute *t*-values as presented in Table 4.

Results from Table 4 demonstrate that all the paths attained *t*-value are higher than the cut-off point for a significance level of 1 percent, that is, 2.58. This implies that all the paths in the model have a strong effect on cost performance. The highest *β* value is 0.718 for contractor's site management related factors. This most significant construct (group of factors) influences critically in affecting cost performance of construction projects.

### 5.3. Model Validation

The developed model is validated to check its usefulness. The validation is carried out by checking the stability of the model through calculating adequacy of sample size with power analysis test. Power analysis (1 − *β*) test is to check the stability of the model's parameters with the sample size used for the analysis [25]. It is to confirm whether the sample size used is sufficient for generating a stable model. The test is conducted by calculating the power of the model through G*Power 3.1.2 software package [38, 39]. Input parameters required for the software are at significance level (*α*) of the test, sample size (N) of the study and effect size (ES) of the population. Effect size is calculated using Cohen et al. [34] equation as below:

(1) $$\begin{array}{c} \text{Effect}\;\text{Size}=\;\frac{R^{2}}{1-R^{2}}, \end{array}$$

where *R* ^2^ is the coefficient of determination.

**Figure 3:**
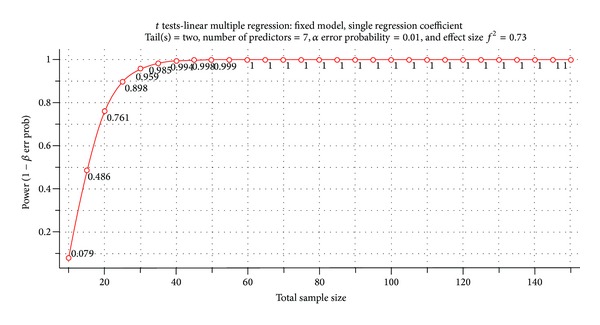
Generated power analysis.

Input parameters for this study are significance level as 0.01 (i.e., 99% of confidence level), sample size (N) as 144, and effect size (ES) as 0.73. The generated values of power analysis for various sample sizes are shown in Figure 3. Figure 3 indicates that the power of the overall model increases as the number of samples size increases. It achieved 100% power at sample size of 50 since this study used 144 samples and it is obvious that it is more adequate for achieving substantial power.

## 6. Conclusion

This study highlighted the cost performance in the construction project affected by various inhibiting factors. These factors are grouped and modelled into 7 categories in SmartPLS software where it was analyzed for assessing the effect on cost performance. Major conclusions drawn from this study are as follows.

- 29 inhibiting factors have a strong effect on cost performance, major conclusion drawn from study.
- *R* ^2^ value of the model is more than 0.26 and classified as a good model where it has substantial degree of explained variance of cost performance by inhibiting factors.
- The sample size of 144 involved in the study was adequate and validated through power analysis test.
- Most significant category of inhibiting factors affecting cost performance in the construction industry is Contractor's site management.

In contractor site management group there are 7 factors which are significant for the contractors to give more emphasis for achieving successful completion of the projects undertaken by them.

## Figures and Tables

**Table 1:** Characteristics of the respondents.

Characteristic	Frequency	Percentage	Cumulative percentage
Experience			
0–5 Years	23	16	16
6–10 Years	23	16	32
11–15 Years	30	20.8	52.8
16–20 Years	15	10.4	63.2
>20 Years	53	36.8	100
Education Level			
BE	110	76.4	76.4
BSc	8	5.6	81.9
Diploma	3	2.1	84.0
MBA	1	0.7	84.7
ME	3	2.1	86.8
MSc	17	11.8	98.6
PhD	2	1.4	100
Working Position			
Executives (directors)	50	34.72	34.72
Managerial personnel	35	24.31	59.03
Engineering staff	54	37.50	96.53
Quantity surveying personnel	5	3.47	100

**Table 2:** Results of measurement model evaluation.

	First iteration				Final iteration			
	Loading	AVE	CR	Alpha	Loading	AVE	CR	Alpha
CSM01	0.639	0.462	0.870	0.879	0.635	0.537	0.872	0.835
CSM02	0.560				Omitted			
CSM03	0.515				Omitted			
CSM04	0.844				0.844			
CSM05	0.815				0.826			
CSM06	0.641				0.651			
CSM07	0.758				0.769			
CSM08	0.585				0.637			
DDF01	0.772	0.467	0.795	0.874	0.806	0.640	0.873	0.852
DDF02	0.401				0.553			
DDF03	0.873				0.921			
DDF04	0.331				Omitted			
DDF05	0.839				0.870			
FIN01	0.596	0.459	0.832	0.815	0.571	0.533	0.816	0.754
FIN02	0.624				0.633			
FIN03	0.604				Omitted			
FIN04	0.518				Omitted			
FIN05	0.813				0.807			
FIN06	0.846				0.867			
ICT01	0.867	0.786	0.917	0.880	0.867	0.786	0.917	0.880
ICT02	0.912				0.912			
ICT03	0.881				0.881			
LAB01	0.861	0.577	0.871	0.828	0.861	0.577	0.871	0.828
LAB02	0.793				0.793			
LAB03	0.778				0.778			
LAB04	0.726				0.726			
LAB05	0.617				0.617			
MMF01	0.795	0.625	0.866	0.821	0.795	0.625	0.866	0.821
MMF02	0.909				0.909			
MMF03	0.575				0.575			
MMF04	0.842				0.842			
PMCA01	0.539	0.444	0.757	0.702	Omitted	0.515	0.757	0.737
PMCA02	0.626				0.590			
PMCA03	0.812				0.848			
PMCA04	0.658				0.692			

**Table 3:** Results of cross loading.

	CSM	DDF	FIN	ICT	LAB	MMFM	PMCA
CSM01	**0.635**	0.389	0.165	0.479	0.396	0.176	0.460
CSM04	**0.844**	0.383	0.384	0.414	0.305	0.368	0.510
CSM05	**0.826**	0.465	0.430	0.456	0.323	0.208	0.480
CSM06	**0.651**	0.191	0.365	0.460	0.407	0.480	0.465
CSM07	**0.769**	0.402	0.313	0.280	0.246	0.172	0.433
CSM08	**0.637**	0.472	0.569	0.520	0.454	0.456	0.629
DDF01	0.304	**0.806**	0.228	0.346	0.255	−0.032	0.519
DDF02	0.351	**0.553**	0.388	0.284	0.194	0.175	0.432
DDF03	0.477	**0.921**	0.366	0.505	0.443	0.305	0.715
DDF05	0.459	**0.870**	0.273	0.443	0.462	0.321	0.578
FIN01	0.286	0.081	**0.571**	0.191	0.440	0.293	0.219
FIN02	0.115	0.011	**0.633**	0.040	0.382	0.194	0.250
FIN05	0.401	0.397	**0.807**	0.298	0.474	0.171	0.452
FIN06	0.346	0.235	**0.867**	0.272	0.561	0.551	0.393
ICT01	0.548	0.394	0.285	**0.867**	0.537	0.511	0.553
ICT02	0.472	0.512	0.292	**0.912**	0.518	0.407	0.587
ICT03	0.514	0.404	0.295	**0.881**	0.498	0.471	0.646
LAB01	0.289	0.323	0.515	0.479	**0.861**	0.423	0.496
LAB02	0.541	0.387	0.574	0.468	**0.793**	0.503	0.497
LAB03	0.357	0.408	0.433	0.481	**0.778**	0.411	0.400
LAB04	0.450	0.473	0.520	0.487	**0.726**	0.561	0.536
LAB05	0.246	0.318	0.498	0.238	**0.617**	0.267	0.276
MMF01	0.250	0.135	0.354	0.280	0.357	**0.795**	0.218
MMF02	0.314	0.256	0.397	0.466	0.501	**0.909**	0.401
MMF03	0.470	0.280	0.390	0.503	0.490	**0.575**	0.405
MMF04	0.389	0.293	0.408	0.512	0.506	**0.842**	0.401
PMCA02	0.244	0.577	0.321	0.362	0.245	0.015	**0.590**
PMCA03	0.591	0.547	0.348	0.571	0.439	0.322	**0.848**
PMCA04	0.456	0.412	0.405	0.528	0.558	0.552	**0.692**

**Table 4:** Path coefficient with *t*-values for the structural model.

		Path coefficient (*β*)	*t*-value
CSM	Contractor's Site Management Related Factors	−0.718	49.43*
DDF	Design and Documentation Related Factors	0.194	11.59*
FIN	Financial Management Related Factors	0.193	14.80*
ICT	Information and Communication Related Factors	0.145	9.43*
LAB	Human Resource (Workforce) Related Factors	0.298	21.82*
MMFM	Nonhuman Resource Related Factors	0.043	4.01*
PMCA	Project Management and Contract Administration Related Factors	0.102	3.55*

*P < 0.01.

## References

[1] Angelo WJ, Reina P Megaprojects need more study up front to avoid cost overruns. http://flyvbjerg.plan.aau.dk/News%20in%20English/ENR%20Costlies%20150702.pdf.

[2] Meng X (2012). The effect of relationship management on project performance in construction. *International Journal of Project Management*.

[3] Chang AS-T (2002). Reasons for cost and schedule increase for engineering design projects. *Journal of Management in Engineering*.

[4] Žujo V, Car-Pušic D, Brkan-Vejzović A (2010). Contracted price overrun as contracted construction time overrun function. *Technical Gazette*.

[5] Gupta N Avoiding time and cost overruns in the construction of Rohtang tunnel. http://www.idsa.in/idsacomments/AvoidingTimeandCostOverrunsintheConstructionofRohtangTunnel_ngupta_141209.

[6] Lee J-K (2008). Cost overrun and cause in Korean social overhead capital projects: roads, rails, airports, and ports. *Journal of Urban Planning and Development*.

[7] Olatunji OA (2008). A comparative analysis of tender sums and final costs of public construction and supply projects in Nigeria. *Journal of Financial Management of Property and Construction*.

[8] Apolot R, Alinaitwe H, Tindiwensi D An investigation into the causes of delay and cost overrun in Uganda’s public sector construction projects.

[9] Khamidi MF, Khan WA, Idrus A The cost monitoring of construction projects through earned value analysis.

[10] Endut IR, Akintoye A, Kelly J Cost and time overruns of projects in Malaysia. http://www.irbnet.de/daten/iconda/CIB10633.pdf.

[11] Rahman IA, Memon AH, Azis AAA, Nagapan S, Latif QBI Time and cost performance of costruction projects in southern and cenrtal regions of Penisular Malaysia.

[12] Byrne BM (2010). *Structural Equation Modeling with AMOS Basic Concepts, Applications, and Programming*.

[13] Hair JF, Ringle CM, Sarstedt M (2011). PLS-SEM: indeed a silver bullet. *Journal of Marketing Theory and Practice*.

[14] Kaming PF, Olomolaiye PO, Holt GD, Harris FC (1997). Factors influencing construction time and cost overruns on high-rise projects in Indonesia. *Construction Management and Economics*.

[15] Koushki PA, Al-Rashid K, Kartam N (2005). Delays and cost increases in the construction of private residential projects in Kuwait. *Construction Management and Economics*.

[16] Enshassi A, Al-Najjar J, Kumaraswamy M (2009). Delays and cost overruns in the construction projects in the Gaza Strip. *Journal of Financial Management of Property and Construction*.

[17] Nawaz T, Shareef NA, Ikram AA (2013). Cost performance in construction industry of Pakistan. *Industrial Engineering Letters*.

[18] Park YI, Papadopoulou TC (2012). Causes of cost overruns in transport infrastructure projects in Asia: their significance and relationship with project size. *Built Environment Project and Asset Management*.

[19] Tenenhaus M, Vinzi VE, Chatelin Y-M, Lauro C (2005). PLS path modeling. *Computational Statistics and Data Analysis*.

[20] Ringle CM, Wende S, Will S SmartPLS 2.0 (M3) Beta. http://www.smartpls.de.

[21] Henseler J, Ringle CM, Sinkovics RR (2009). The use of partial least squares path modeling in international marketing. *Advances in International Marketing*.

[22] Vinzi VE, Trinchera L, Amato S, Vinzi VE, Chin WW, Henseler J, Wang H (2010). PLS path modeling: from foundations to recent developments and open issues for model assessment and improvement. *Handbook of Partial Least Squares*.

[23] Hair JF, Sarstedt M, Ringle CM, Mena JA (2012). An assessment of the use of partial least squares structural equation modeling in marketing research. *Journal of the Academy of Marketing Science*.

[24] Gotz O, Liehr-Gobbers K, Krafft M, Vinzi VE, Chin WW, Henseler J, Wang H (2010). Evaluation of structural equation models using the Partial Least Squares (PLS) approach. *Handbook of Partial Least Squares*.

[25] Chin WW, Marcoulides GA (1998). The partial least squares approach to structural equation modeling. *Modern Methods for Business Research*.

[26] Hair JF, William CB, Barry JB, Anderson RE (2010). *Multivariate Data Analysis*.

[27] Hulland J (1999). Use of Partial Least Squares (PLS) in strategic management research: a review of four recent studies. *Strategic Management Journal*.

[28] Fornell C, Larcker DF (1981). Evaluating structural equation models with unobservable variables and measurement error. *Journal of Marketing Research*.

[29] Litwin MS (1995). *How to Measure Survey Reliability and Validity*.

[30] Barclay D, Thompson R, Higgins C (1995). The Partial Least Squares (PLS) approach to causal modeling: personal computer adoption and use as an illustration. *Technology Studies*.

[31] Akter S, Ambra JD, Ray R An evaluation of PLS based complex models: the roles of power analysis, predictive relevance and GoF index.

[32] Lleras C (2005). Path analysis. *Encyclopedia of Social Measurement*.

[33] Cohen J (1988). *Statistical Power Analysis for the Behavioral Sciences*.

[34] Cohen J, Cohen P, West SG, Aiken LS (2003). *Applied Multiple Regression/Correlation Analysis for the Behavioral Sciences*.

[35] Aibinu AA, Al-Lawati AM (2010). Using PLS-SEM technique to model construction organizations’ willingness to participate in e-bidding. *Automation in Construction*.

[36] Davison AC, Hinkley DV (1997). *Bootstrap Methods and Their Application*.

[37] Efron B, Tibshirani RJ (1993). *An Introduction to the Bootstrap*.

[38] Erdfelder E, FAul F, Buchner A, Lang A-G (2009). Statistical power analyses using G*Power 3.1: tests for correlation and regression analyses. *Behavior Research Methods*.

[39] Faul F, Erdfelder E, Lang A-G, Buchner A (2007). G*Power 3: a flexible statistical power analysis program for the social, behavioral, and biomedical sciences. *Behavior Research Methods*.

